# Developing a digital data platform for surveillance of food and water-borne pathogens in North East India: insight for public health advocacy

**DOI:** 10.3389/fpubh.2024.1422373

**Published:** 2024-08-26

**Authors:** Samaresh Das, Harleen Kaur, Subhankar Mukherjee, Manas Chakraborty, Raja Gupta, Shalony Roy, Indranil Ganguly, Tapan Majumdar, Karma Gyurmey Dolma, Pallab Sharma, Suranjana Chaliha Hazarika, Dalem Modi, Thandavarayan Ramamurthy, Madhuchhanda Das

**Affiliations:** ^1^Centre for Development of Advanced Computing (CDAC), Kolkata, India; ^2^ICMR-National Institute for Research in Digital Health and Data Science (ICMR-NIRDHDS), New Delhi, India; ^3^Department of Microbiology, Agartala Government Medical College, Agartala, India; ^4^Department of Microbiology, Sikkim Manipal Institute of Medical Sciences (SMIMS), Sikkim Manipal University, Gangtok, India; ^5^ICMR Regional Medical Research Centre (RMRC), Dibrugarh, India; ^6^Department of Microbiology, Gauhati Medical College and Hospital, Guwahati, India; ^7^Department of Microbiology, Bankin Pertin General Hospital and Research Institute, Pasighat, India; ^8^ICMR-National Institute of Cholera and Enteric Diseases (NICED), Kolkata, India; ^9^Indian Council of Medical Research, New Delhi, India

**Keywords:** Food-borne, surveillance, NE India, ICMR, public health

## Abstract

Robust digital infrastructure is vital and the need of the hour, especially in the healthcare sector, for real-time data generation, analysis, and quick decision-making. Food- and water-borne illnesses represent a prominent cause of morbidity and mortality worldwide. India, a developing nation with diverse cultures and food practices, poses a high risk of food-borne diseases and outbreaks, yet is often underreported and ineffectively researched. Also, the unique socio-economic and environmental factors of the Northeast (NE) region contribute to the high burden of food-borne diseases. To address these trepidations, the Indian Council of Medical Research (ICMR) has undertaken a study for the surveillance of food-borne pathogens in NE India. The present study focuses on the development of a digital database system for the systematic surveillance of foodborne disease outbreaks, aiming to address the gaps in traditional surveillance methods and improve disease detection and response capabilities. The digital system integrates mobile applications, web-based platforms, and advanced analytics tools to enable real-time data collection, dissemination, and analysis of food-borne illness data. Additionally, the secure and scalable nature of the system enhances data accuracy and accessibility, making it a valuable tool for enhancing food-borne disease surveillance efforts in resource-constrained settings.

## 1 Introduction

Digital health care and data management have advanced enormously in the last few years. The COVID-19 pandemic showed the importance of real-time data management during a public health emergency. A food-borne outbreak requires quick investigation and data analysis to control emergencies. Furthermore, large-scale surveillance studies need a digital database to identify the early signal of an outbreak. World Health Organization (WHO) and the US Centre for Disease Control and Prevention (CDC) systematically report food-borne outbreaks and diseases every year (1, 2). Globally, about 600 million people are affected due to food-borne infections and diarrheal diseases causing 420,000 deaths every year (3). Interestingly, more than 60% of emerging and re-emerging pathogens, which are propagated through food and water to humans originated from animals and the environment (4). The gastrointestinal tracts of domestic and farm animals (poultry, cattle, swine, sheep, and goats) are host to a large number of zoonotic pathogens, many of which are shed in their feces. Meat and meat products, milk, and egg is often contaminated during unhygienic processing environments and through food handlers. The most prevalent enteric pathogens linked to numerous food and water-borne outbreaks are *Campylobacter*, *Salmonella*, and *Shigella* which are also becoming resistant to many antibiotics thereby limiting the treatment options (5). In addition to these widely recognized viruses and bacteria, fungal pathogens are also known to cause food-borne illnesses typically due to secondary metabolites such as toxins in vulnerable and immunocompromised individuals (6).

Susceptibility to food and water-borne infections, which are a major source of morbidity and mortality is high, especially in developing nations like India. Although very scanty data is available due to the focal and self-limiting nature of the disease, they are often not notified to the health authorities. Over the past 29 years (1980–2009) a total of 37 outbreaks have occurred due to food poisoning affecting 3,485 persons (7), which is just the tip of the iceberg. In the absence of systematic data on food and water-borne diseases, it is difficult to plan for a strategic control program. Given these lacunae, ICMR has initiated comprehensive food and water-borne disease pathogen surveillance in NE India (8). Unique cultural diversity and food habits of NE people coupled with the relatively high prevalence of Hepatitis A and E is the stimulation for an organized study (8, 9). A digital database developed to collect real-time data and to identify early outbreak indication, this paper describes the comprehensive process, methodology, and roles of different users in developing and implementing a web-based data retrieval, analytics, and repository system. This digital and analytical platform will bridge the gap in monitoring food-borne illnesses in India by creating a comprehensive, real-time, and data-driven surveillance system.

The knowledge of disease processes is not the only factor that determines the quality of medical care, but also how practitioners organize, interpret, and diagnose clinical data (10). Henceforth, the secure and user-friendly digital platform in the present study is groundbreaking as it is a unique platform in NE India, addressing a significant gap in food safety monitoring in this region. Unlike other tools, this platform integrates data from diverse sources such as markets, hospitals, and outbreak sites, enabling regional and cultural specificity for real-time surveillance. Additionally, its scalable and secure nature bridges the gaps in traditional surveillance methods while the information gathered through this study will lay hold of the true magnitude of the disease and help the policymakers monitor outbreaks, evaluate public health interventions, build policy, and gauge the progress in disease prevention.

The study objectives cover the identification of major pathogens causing food and water-borne diseases, documenting regional and seasonal variations in diarrheal diseases, identifying outbreak sources, and performing genotyping and antibiotic sensitivity tests on bacterial pathogens. This also seeks to determine risk factors associated with sporadic and outbreak infections, contributing to evidence-based strategies for preventing food-borne diseases.

## 2 Materials and methods

**Figure 1:**
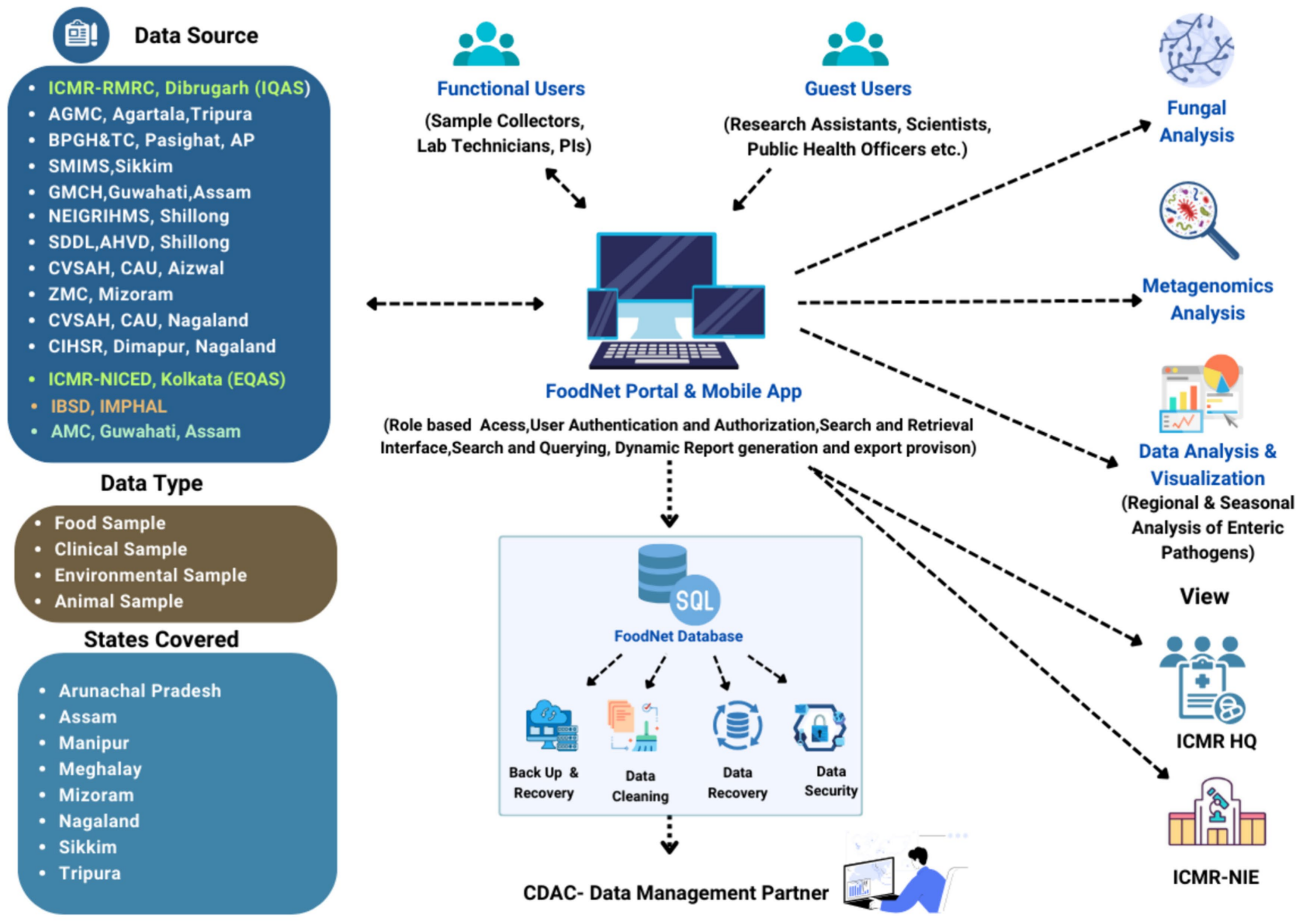
Flow diagram depicting the process cycle of the FoodNet platform (https://icmrfoodnet.in/).

The ICMR initiated the Task Force project in 2020 to investigate the food and waterborne infections covering the NE region of the country. The first phase of the study was initiated in four states, i.e., Assam (Dibrugarh and Guwahati), Arunachal Pradesh, Sikkim, and Tripura, which has systematically been expanded to the other NE regions (Manipur Mizoram, Meghalaya, Nagaland) of India in Phase II (8). A total of 12 centres, including nine medical and three veterinary centres were initiated to cover the entire NE region. The details of the complete network are given in Supplementary Figure 1. The ICMR headquarters coordinated planning, funding, and logistical support for regional centers. ICMR-National Institute of Cholera and Enteric Diseases (ICMR-NICED), Kolkata managed External Quality Assurance (EQAS) and training, ICMR-National Institute of Epidemiology (ICMR-NIE) focused on outbreak training, and CDAC, Kolkata served as the data management partner to develop the integrated digital platform, including a mobile app for data collection and a web-based data repository. Figure 1 illustrates the detailed and stepwise process flow to develop an integrated and extensive system of such kind as proposed in this study.

### 2.1 Strategizing the development process

#### 2.1.1 Determine the scope and objectives

The formulation of standardized data collection has become the need of the hour in good clinical data management. The risk that food-borne pathogens pose for public health, which can also vary in their signs and symptoms, susceptibility profiles, and treatment response has become the ground for systematic clinical data management. Data mining, a technique increasingly employed in medical practices is data dependent maintained within the data management system for its execution. Simultaneously, the progression of the disease can be sought within the affected population by analyzing the trends and patterns of the outbreak. Nonetheless, the inadequate or absence of such systems especially in extensive and multicentered studies could lead to manual and traditional data processing potentially compromising the research process and its outcome. In clinical studies, especially involving human interventions, delayed or erroneous data may pose a risk to patient safety. Furthermore, for national priority projects, systematic implementation of the initiatives within the target population could be achieved with the extensive digital system.

#### 2.1.2 Determine the stakeholders and their roles

The development of such a large automated system involves various stakeholders, bringing in their unique expertise and perspectives. Primary stakeholders include healthcare experts, database and mobile app developers, and data managers thereby providing domain knowledge and insights in the development process. ICMR-Technical Advisory Committee (TAC) has approved the study protocol and Case Report Form (CRF) ensuring regulatory compliance and standards. Field workers from each centre were responsible for the collection of food samples from markets and clinical samples from hospitals through the physical documentation process or the FoodNet mobile app. The information collected was then integrated into the centralized web-based repository using the data collector’s login credentials. Further, it followed the systematic process of verification and approval of sample information, sample assignment to respective laboratory technicians, and subsequent upload of bacterial characterization results after the final approval of each principal investigator (PI) i.e., the centre administrator. Those samples with enteric pathogens are further sent to ICMR-NICED and ICMR-Regional Medical Research Centre (ICMR-RMRC), Dibrugarh to confirm test results further and upload them in the repository. To facilitate this process from data collection to data submission, the developers oversaw the design and implementation of the technical infrastructure for both the web app and mobile app. A comprehensive e-Lab module has been developed for lab technicians working at participating centres and ICMR-RMRC and ICMR-NICED. This module is a repository for storing granular-level test results from various laboratory tests conducted during the surveillance project. This involved prioritizing data security, adhering to both functional and non-functional requirements, and ensuring the seamless operation of the entire system. Each user role in the system has distinct responsibilities and access levels, confirming that data collection, validation, approval, and analysis are carried out efficiently and securely to achieve the study objectives.

**Figure 2:**
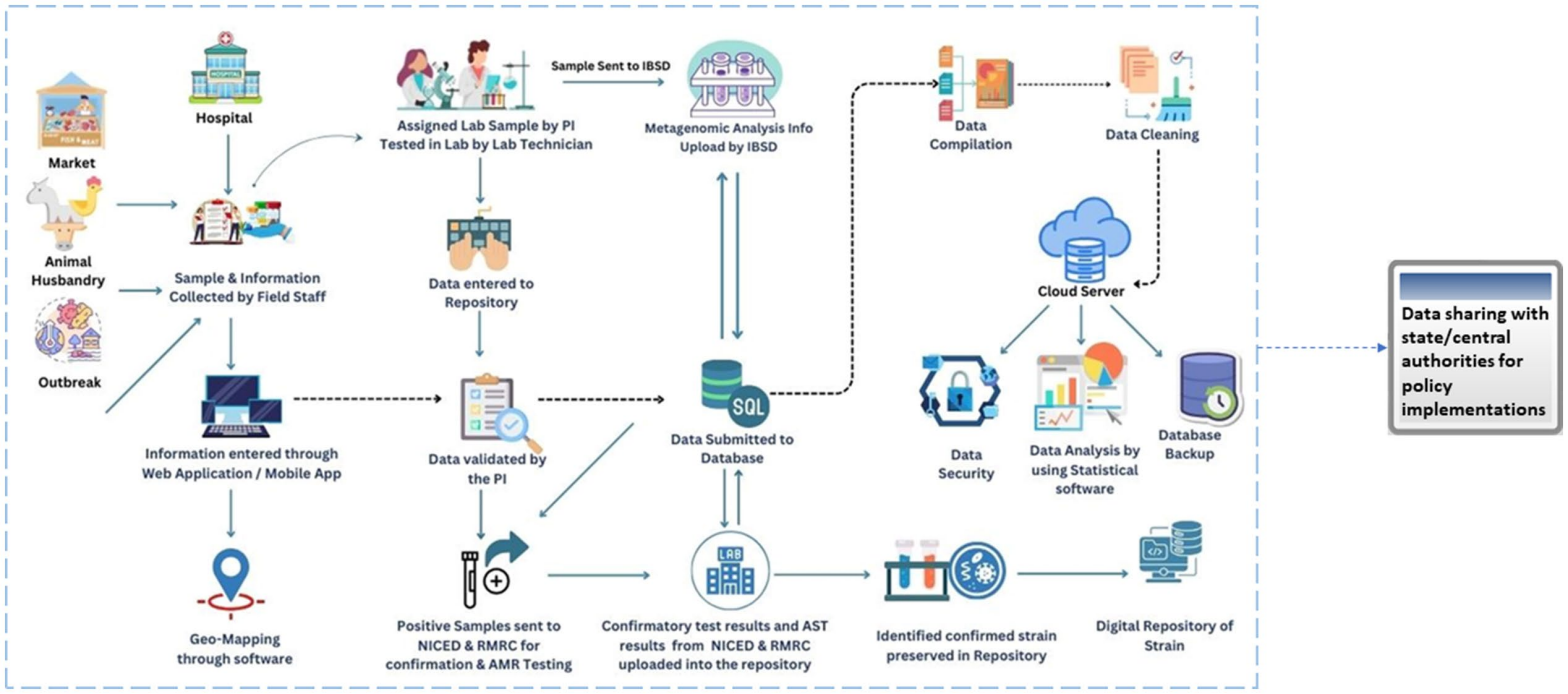
Flow diagram depicting the workflow of the FoodNet platform.

Figure 2 illustrates the active involvement of all the stakeholders, especially the local community involvement ensuring that the data collected is tailored to the ground realities and regional needs. Healthcare providers contribute clinical knowledge, improving the detection of trends and patterns in disease prevalence whereas food safety authorities ensure compliance with regulatory standards, enhancing data reliability. Hence, the system aims to uncover trends, fluctuations, and associations between specific food-borne illnesses, foods, and habits. By identifying causes and contamination sources, the platform significantly contributes to understanding food-borne disease incidence over time.

#### 2.1.3 Design a CRF

In collaboration with the multidisciplinary team, a comprehensive data collection protocol was developed prioritizing patient privacy and data security in adherence to ethical guidelines and regulatory standards outlined by the ICMR and other relevant authorities. The CRF records the data from the market surveillance samples, clinical samples from hospital surveillance, food animal samples, animal food products, animal living environment samples from piggery farms, poultries, dairy farms, clinical samples, and leftover food, environmental and food handlers’ samples from outbreak investigation. CRF was designed comprehensively to encompass diverse data points, ensuring that it captures all pertinent information related to food-borne pathogens while being user-friendly for healthcare professionals. Inclusion and exclusion criteria for the hospital and market surveillance data were also taken into consideration as given in Table 1. Details on the data elements for each component, i.e., hospital, market, poultry/farm/slaughterhouse surveillance, and outbreak investigation are given in Supplementary Tables 2–5, respectively. The simplified data input process, facilitated by user-friendly forms and data entry prompts, also minimizes errors during data entry. For case definitions in the study, we have considered guidelines of the Integrated Diseases Surveillance Project (IDSP), Ministry of Health and Family Welfare (MoHFW), and Government of India (GoI), the details of which are given in Table 2. The definitions given by WHO/CDC may be adapted as and when required, e.g., IDSP definition is not available or if the situation demands.

**Table 1:** Inclusion and exclusion criteria for hospital and market surveillance.

Inclusion criteria	Hospital surveillance: any diarrhea, or vomiting case more than 1 year of age with a history of suspected food intake.
	Market surveillance: a list of food items (Supplementary Table 1) only be collected from identified markets.
	Outbreak investigation: any outbreak confirmed by IDSP or as per the outbreak definition. Any suspected food, food animal, water, environmental, food handlers, and human samples.
Exclusion criteria	Infants below the age of 1 year. Patients/individuals (carriers) who did not consent to give samples.

**Table 2:** Case definitions as per IDSP.

Food-borne disease outbreak	The occurrence of 2 or more cases of a similar illness resulting from ingestion of a common food or the observed number of cases of a particular disease exceeds the expected number. These can be confirmed (when at least one causal agent is identified) or suspected (based on clinical and epidemiological information). Although most cases are sporadic, these diseases draw attention to themselves due to outbreaks, thorough investigation of which can help in identifying control measures (20).
Diarrhea	Passage of 3 or more loose watery stools in the past 24 h [with or without vomiting; modified case definitions of the P form under IDSP (January 2017)].
Food-borne disease	The term Foodborne diseases, including foodborne intoxications and foodborne infections, covers illnesses acquired through consumption of contaminated food, and are also frequently referred to as food poisoning (20).

#### 2.1.4 Integrated data collection

On-site data collection was done by the designated data collectors from each participating centre. Data collection was done either in real-time through the Food-Net mobile app or using the paper-based CRF. Data collected in paper form was later uploaded by the data collector to the web-based repository. In case of unavailability of the internet during the data collection, provision to store the data in the local mobile storage has been provisioned. The data synchronization with a centralized web repository occurs upon connection to the internet. This ensures uniform and accurate data across all users and devices. The app additionally eases the data collection in offline environments where internet access is restricted, and the data synchronization occurs upon connection to the internet. This real-time data collection also helped to develop geo-mapping of food-borne infections and outbreaks. The FoodNet mobile app is also available in the Google Play store.^1^

#### 2.1.5 Database design considerations

Based on the requirements, a system architecture was designed for mobile as well as web applications, outlining the major components, data flow, and interactions between different modules. Choosing the appropriate data model also plays a key role. Henceforth, the relational data model was preferred due to its flexibility and scalability. The database schema was designed to store diverse surveillance data, test results, and user information. Sample details from targeted markets, hospitals, and outbreak locations were defined in the database as well as CRF layouts designed for data entry. Establishing relationships to integrate different modules (data collection, lab testing, data validation, etc.) was done to ensure seamless data flow and communication between modules. The schema was also normalized to optimize storage, minimize data redundancy, and ensure data integrity, and application programming interface (APIs) or middleware components were implemented to facilitate synchronization between different modules of the mobile and web applications. The choice of technologies, frameworks, and tools was made based on the study’s scalability, security, and performance requirements.

#### 2.1.6 Developing the data management system

This process involves the translation of the designed prototype into a functional and user-friendly system for the investigation of food-borne pathogen outbreaks. The complete architecture was crafted using the Django framework with the MySQL database that stores all the collected data in the defined schema tables along with the support for complex queries. In the front end, HTML, CSS, and JavaScript were used. FoodNet mobile app was built on Android Studio using JAVA programming language. Hosting was facilitated using Apache and Nginx web servers. Hence, the complete digital system was crafted using the open-source database management system, web, and user interface design.

#### 2.1.7 Training and capacity building

Comprehensive training sessions, both offline and online, were conducted by CDAC, Kolkata for all the users involved, i.e., field workers, lab technicians, healthcare workers, researchers, and data-providing centres. The training programs were tailored to meet the diverse needs of users with different functional roles including hands-on sessions to become familiar with the digital platform. Additionally, the skill levels of users are considered during the training to ensure effective learning. Numerous workshops were conducted where participants received thorough briefings for real-time sample collection from markets, hospitals, communities, and outbreak locations, as well as submitting the results into the repository. Individual sessions were also conducted to resolve the queries, especially of field workers. Additionally, the complete team was provided with a detailed user manual that outlines the process flow of the system, enhancing their understanding and troubleshooting capabilities. Through these comprehensive training sessions and ongoing support, users were equipped to effectively utilize digital tools and interpret data, facilitating timely interventions in the field of food-borne surveillance.

#### 2.1.8 CRF tracking and field testing

Once sample information is uploaded into the system, either through the FoodNet Mobile App or manually via paper-based CRF, a unique FoodNet Sample ID is automatically generated. This ID is distinct across all surveillance types and is formed by combining abbreviated codes representing the state name, district name, sample category, and sequence number. By referencing the Food-Net Sample ID, one can readily discern meta-information about the sample, such as its collection origin and sample type. This ID serves as the singular identifier for tracking every aspect of a sample, including its source, handler, and bacterial characterization results. While including the sample information or its lab results, certain mandatory fields need to be filled in to proceed to the next segment of the form. At least 100 CRFs containing the data on market and hospital, animal husbandry surveillance, and outbreak investigation were documented for field testing to finalize the CRF. Modifications to the CRF were then made and the CRF was locked after satisfactory feedback from PIs and experts.

#### 2.1.9 Quality control and data validation

Each module was subjected to unit testing to ensure that individual components functioned correctly. Integration tests were conducted to validate that those different modules interacted as expected. Validation criteria and data checks were implemented to ensure the accuracy and reliability of data entered into the system. As a part of security control measures, encryption and access controls were implemented to protect sensitive data. Quality Assurance (QA) procedures were also followed to identify and rectify any defects or issues in the system. All the validation and security checks were carried out pre and post-development process.

#### 2.1.10 Visualizing data dynamics

The web-based application employed a dashboard for dynamic data visualization, including graphs and charts. A role-based user dashboard has been created to cater to different user roles involved in the surveillance project. The dashboard is accessible to stakeholders, including data collectors, lab technicians, and state administrators of the participating centres along with the Institute of Bioresources and Sustainable Development (IBSD). Each role has specific functionalities and access privileges within the system. The main objective of the dashboard was to provide real-time visual representations highlighting trends and patterns in food-borne illnesses and identify specific food associations of food-borne infection in NE India using state-wise data collection reports and bacterial characterization reports generated. Additionally, the dashboard includes the functionality to export the data in PDF and Excel formats for advanced analysis and visualization. The graphical user interface (GUI) was designed to be responsive, ensuring it works well on both desktop and mobile devices.^2^

### 2.2 Maintenance and sustainability

#### 2.2.1 Ensuring up-to-date insights and system scalability

The FoodNet surveillance system utilizes a modular architecture and a microservices approach, where each component such as data collection, storage, analysis, and reporting functions as an independent service. This design ensures that updates or scaling of individual components do not impact overall system performance. The system also leverages the vertical scalability feature of the application hosting infrastructure by adjusting the resources such as the Central Processing Unit (CPU), Random Access Memory (RAM), and storage according to the changing needs of the applications or services. This flexibility manages the workloads efficiently without experiencing downtime or performance degradation.

By capturing the most recent insights, the database stays a relevant and valuable resource, ensuring that end users and healthcare professionals have the most up-to-date information to guide their patient care decisions.

#### 2.2.2 Backup and disaster recovery

Regular data backups and a disaster recovery plan are in place to ensure data integrity and availability in case of system failures or cyberattacks. Backups are stored in offsite locations to prevent data loss in the event of disasters such as server failures or physical damage to the hosting environment. Backup data is also encrypted to prevent unauthorized access during storage and transmission to offsite locations.

#### 2.2.3 Security and performance monitoring

Each user role in the system has distinct responsibilities and access levels, ensuring that data collection, management, and analysis are carried out efficiently and securely to achieve the project’s objectives. All data exchanged between the web platform and users is encrypted using hypertext transfer protocol secure (HTTPS) to protect data in transit. A strong secure hash algorithm 2 (SHA2) with a 2048-bit encryption-based secure sockets layer (SSL) certificate has been installed in all kinds of secured communication. Data input from users is validated and sanitized to prevent common security vulnerabilities such as structured query language (SQL) injection and cross-site scripting (XSS). Access control lists (ACLs) are used to control and restrict access to sensitive resources and data within the application. Security training and awareness programs were also conducted for all users and developers to promote best security practices.

#### 2.2.4 Maintaining the system performance

Regular communication with participating centers at regular intervals facilitates resolving any issues that arise quickly, which is essential to the database’s functioning. Whether correcting technical malfunctions, data discrepancies, or security concerns, a timely approach to issue resolution is taken to provide continuous access to essential information. Immediate response increases user trust and preserves the database’s credibility as a credible resource.

## 3 Discussion

Zoonotic diseases are on the rise globally causing outbreaks of systematic infectious diseases and primarily one of the causes of morbidity and mortality. Over the past 145 years, the identification of most agents responsible for food-borne illness has been a progressive journey, beginning with the ground-breaking research of Robert Koch that led to the discovery of causative agents of anthrax, tuberculosis, and cholera (11). Over time, however, the research for the identification of new agents of the pathogens has been very challenging to observe in isolates and cultures. The complexity of the disease has thus necessitated the development of the surveillance system beyond the confines of manual data management specifically in developing countries like India with the population growing at an exponential rate. Data management being a crucial component, especially in multicentric studies, various surveillance systems and networks play an important role in tracking, monitoring, and understanding the disease. For example, Listiwiki is a database dedicated to the food-borne pathogen *Listeria Monocytogenes* (12). Food and environment-associated anti-microbial resistance (FEAMR) provides information on the surveillance of AMR in food, environment, and other non-clinical samples globally (13). Another such system includes a novel database that analyses Twitter data and National Outbreak Reporting System (NORS) data for detecting and evaluating food-borne illnesses in the United States (14). The OzFoodNet network, initiated by the Australian Government Department of Health in 2000, was established for national collaboration in the investigation of food-borne diseases. Within this framework, epidemiologists delve into outbreaks of enteric infection, conduct studies on illness burdens, and coordinate national inquiries into outbreaks of food-borne diseases (FBDOs) (15, 16). The Foodborne Disease Outbreak Surveillance System (FDOSS), CDC’s program is another such system for collecting and reporting data about foodborne disease outbreaks in the United States. WHO Global Food-borne Infections Network (GFN) is a network of institutions to detect, respond, and prevent food-borne and other enteric infections (17). Similarly, the European Centre for Disease Prevention and Control (ECDC) collects and analyses data on food-borne and water-borne diseases in the European Union (EU) through its surveillance system, including the European Surveillance System (TESSy) and the food- and water-borne Diseases and Zoonoses Network (FWD-Net) (18). Global Enteric Multi-Centre Study (GEMS) is a large-scale study funded by the Bill and Melinda Gates Foundation that aims to estimate the burden and causes of childhood diarrhea in Africa and Asia (19). Apart from these global surveillance networks, India despite possessing a robust public health infrastructure and disease burden estimation programs, it lacks a specific focus on individual disease prevention and control. For instance, IDSP reports only food-borne outbreaks. Whereas, the Food Safety and Standards Authority of India (FSSAI) established by GoI ensures the availability of safe food for human consumption. This lacunae and fragmentation have thus made it challenging to integrate the standardized data hindering the understanding of the disease.

The present study manifested the development and implementation of a digital platform for food-borne pathogen surveillance that signifies a critical advancement in addressing the challenges posed by foodborne illnesses, especially in North East India. ICMR FoodNet database quickly generated the list of enteric pathogens identified from the study and their antibiotic resistance profile. Real-time outbreak data, particularly AMR data helped clinicians prescribe the correct antibiotics for the affected patients and helped quick recovery. Identification of the source and the causative organism of the outbreak helped to quick epi curve, spot map, and report generation and dissemination. Food handlers and shopkeepers in the markets were immediately informed once pathogens were identified in their food samples and followed up to prevent the spread of infections. Additionally, the integration of diverse data sources, including clinical records, laboratory reports, and environmental data monitoring, provides a comprehensive view of food-borne illness trends and patterns in the region.

Despite our clinical data management system’s numerous strengths, there remain areas that require improvement. Firstly, ensuring seamless integration of data collection from diverse sources such as markets, hospitals, and outbreak locations posed a significant technical challenge. Harmonizing data formats and protocols across multiple institutions and geographical locations required meticulous planning and coordination. Developing robust encryption mechanisms and user authentication protocols to safeguard sensitive health data was a complex task. Incorporating real-time data collection capabilities through the FoodNet Mobile App necessitated extensive testing and optimization to ensure reliability and usability, especially in remote and resource-constrained areas. Additionally, training personnel at various levels to effectively utilize the digital platform and adapt to new data collection methods posed a challenge. Overcoming these challenges required close collaboration between stakeholders, iterative development cycles, and continuous feedback mechanisms to ensure the platform’s effectiveness in enhancing food-borne disease surveillance and public health response efforts.

### 3.1 Conclusion

Food safety is an important public health issue. In the era of digitalization, epidemic and pandemic, and antimicrobial resistance, real-time data analysis is the utmost need of the hour to control any emergency situation. A developing country like India with a huge population burden, faces a large number of unreported food-borne outbreaks every year, causing the death of under-five children. The gap in monitoring food-borne illnesses in India stems from the lack of systematic surveillance and comprehensive data collection. The digital system offers several advantages, including real-time data collection, analysis, and response, thereby enhancing the timeliness and accuracy of disease surveillance efforts and policy decisions. Leveraging advancements in artificial intelligence (AI) and machine learning (ML) technologies can significantly augment the platform’s capabilities in the future. Enhancing the platform’s interoperability by integration with electronic health records (EHRs) and other public health databases can enrich the dataset with additional contextual information, enabling more comprehensive analyses and actionable insights. Furthermore, the collaborative stakeholder engagement adopted in the entire network enhances the scalability of the system in the long term.

## Data Availability

The raw data supporting the conclusions of this article will be made available by the authors, without undue reservation.

## Footnotes

^1^ https://play.google.com/store/apps/details?id=com.cdackolkata.icmr_foodnet

^2^ https://icmrfoodnet.in/strainrepo/
