# Supplementary material for: Developing a digital data platform for surveillance of food and water-borne pathogens in North East India: insight for public health advocacy
Source: Front Public Health. 2024 Aug 26;12:1422373. doi: 10.3389/fpubh.2024.1422373 (PMC11381271; doi:10.3389/fpubh.2024.1422373)
Supplement: Supplementary file 1 [file Data_Sheet_1.pdf]

## Supplementary Material

# 1 Supplementary Figures and Tables

## 1.1 Supplementary Figures

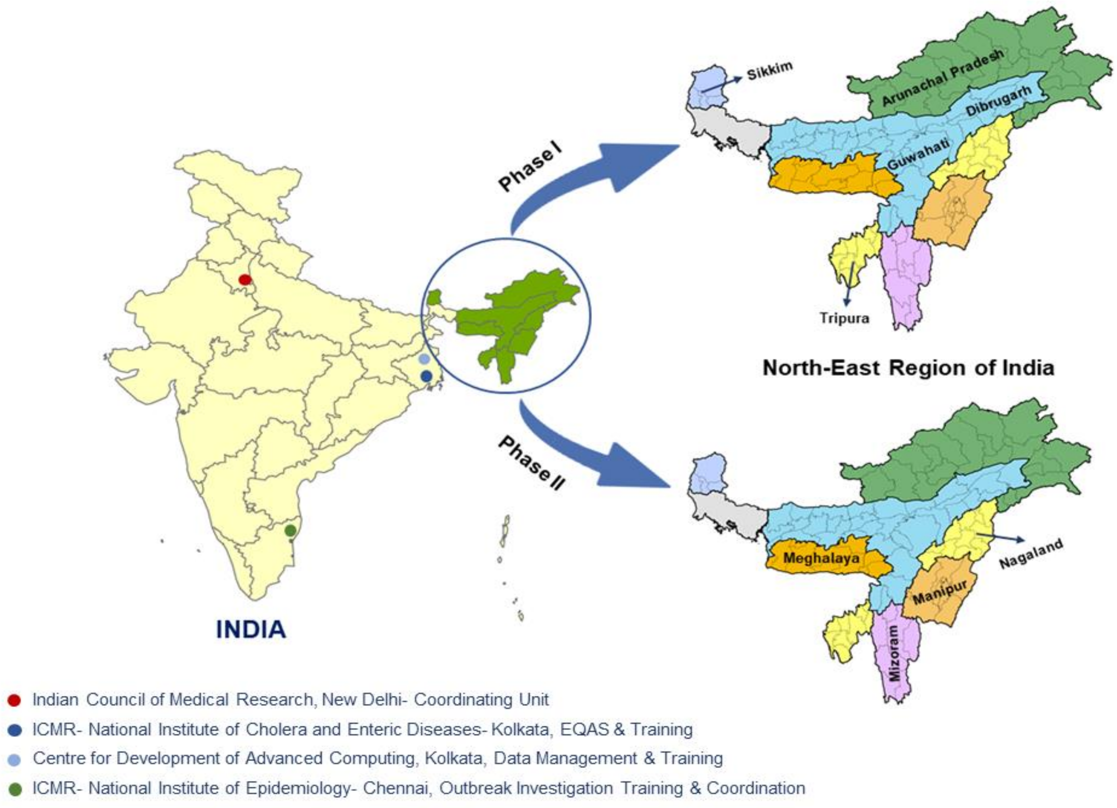


**Supplementary Figure 1.** Research centres from the North-East Region of India involved in the FoodNet surveillance study.

## 1.2 Supplementary Tables

**Supplementary Table 1: List of food items**

| Cooked food (restaurant/street food/ midday meal) (5 items/per category/month) | | Uncooked food (5 items/per category/month) | |
|---|---|---|---|
| Category | Items | Category | Items |
| Non-veg (05) | Beef | Raw/dried meat | Mutton |
| | Chicken | | Chicken |
| | Mutton | | Beef |
| | Pork | | Pork |
| | Fish | | Buffalo |
| | Egg | | Others |
| | Others | Raw/dry fish | Raw fish |
| Rice, flour, pulses (05) | Rice | | Prawn |
| | Chowmein | | Dry fish |
| | Puri/paratha | Milk | Raw milk |
| | Daal | Fruits, vegetables & salads | Whole or cut fruits and vegetables |
| | Beans | Dough | Maida dough |
| | Spinach | | Rice dough |
| | Others | | Dosa/idli batter |
| Milk products & sweets(processed) (05) | Butter | | Others |
| | Cheese | Fermented/ processed/ preserved | Bamboo shoots |
| | Paneer | | Soya bean |
| | Lassi | | Pickles |
| | Curd | | Others |
| | Cake | Environmental | Surface swabs from cutting surfaces/chopping boards |
| | Sweets | | Food handler nail bed swab |
| | Others | | Knife |
| Refrigerated (05) | Ice cream | | Hand wash of food handlers |
| | Pudding | | Skin swab |
| | Milkshake | | Others |
| | | Water | River/pond/stream water |
| | | | Stored water used for vegetable and meat cleaning |

| | | |
|---|---|---|
| | | Tap water |
| | | Ice cream scoop/cone wash |
| | | Others |

| State-specific food items (5 items/ month) | |
|---|---|
| State | Items |
| Arunachal Pradesh | Ekung |
| | Eyup |
| Assam | Pani hamuk |
| | Silk worms |
| | Khar |
| | Ou tenga |
| | Alu pitka |
| Sikkim | Kinema |
| | Gundruk |
| | Chupri |
| | Sidra |
| | Phaley |
| Tripura | Bangoi |
| | Godak |
| Mizoram | Vawksarep |
| | Saum |
| | Daul rep |
| | Rawtuai rep |
| Meghalaya | Buhchiar |
| | Fermented beans (‘tung rymbai) |
| | Fermented fish (‘tung tap) |
| | Blood pulao (jadoh) |
| | Rice cake (kpu maloi) |
| | Fermented bamboo |
| Manipur | Shoots (lung siej) |
| | Fermented bamboo shoots (soibum, soidon) |
| | Fermented soybean (hawaijar) |
| | Fermented fish (ngari) |
| | Local fermented beverage (atingma) |
| | Local pickles |
| Nagaland | Zedicang (fermented mustard leaves extract) |
| | Beurae (colocasi a stem) |
| | Tangcang (fermented soybean) |
| | Eripuka (silk worm) |
| | Kabasuku (fermente d pork fat) |

**Supplementary Table 2:** Overview of data elements of hospital survey

| **CRF NO** | Date: DD/MM/Year | Name of the person completing the form | |
|---|---|---|---|
| | | | |
| **Hospital Name** | | | |
| **Information of the patient** | | Name | |
| | | Age | |
| | | Gender | |
| | | Ethnicity | |
| | | Address & Phone number | |
| **Clinical details** | | Date and time of onset of symptoms | |
| | | Date and time when symptoms stopped | |
| | | **Predominant symptoms** | |
| | | **Symptom** | **Frequency/Duration** |
| | | Diarrhea | |
| | | Blood in stool | |
| | | Vomiting | |
| | | Nausea | |
| | | Fever/feverish | |
| | | Abdominal Pain | |
| | | Others | |
| | | Doctor consulted? *(if yes, provide name and details such as the type of treatment, duration of illness, and names of antibiotics if any)* | |
| | | Date of admission | |
| | | Date of discharge | |
| | | Diagnosis available? | |
| | | **Treatment details** | |
| | | Antibiotic details (if taken) | |
| | | Duration of treatment | |
| | | Outcome | |
| | | Laboratory specimen taken? *(if yes, provide details)* | |
| | | Nature of the specimen | Lab results |
| **Exposure history of the patient** | | Suspect food? | |

| | | | |
|---|---|---|---|
| | Date and time of exposure to an implicated food or event (if known) | | |
| | Source of food | | |
| | Suspect meal, event, place? *(if yes, describe; provide, name, date, address, phone)* contact with people with similar clinical signs and symptoms | | |
| | **Persons attending suspect meal/event** | | |
| | Ill/well | Address & contact no. | |
| | Recent farm visits | | |
| | Contact with animals | | |
| | Working as a food handler | | |
| | Attending or working in a school, Child-care facility, medical facility | | |
| | Chronic illness, immunosuppression, pregnancy | | |
| | Recent changes in medical history, regular medications | | |
| | Allergies, recent immunizations | | |
| | Information on recent travel (domestic and international) | | |
| **Food History** | **Have you eaten any of the following**? | **Amount** | **Source** |
| | Poultry | | |
| | Pork | | |
| | Beef | | |
| | Lamb | | |
| | Chicken | | |
| | Dried smoked meat | | |
| | Crab chutni | | |
| | Dried smoked fish | | |
| | Fish chutni | | |
| | Gungdruk | | |
| | Cauliflower | | |
| | Carrots | | |
| | Green salad | | |
| | Other salads | | |
| | Roast potatoes | | |
| | Fried potatoes | | |
| | Mayonnaise | | |
| | Milk | | |
| | Stored food | | |
| | Ambient | | |
| | Refigerated | | |

| | | | |
|---|---|---|---|
| | Other | | |
| Lab Report | **Baseline Data (0 day)** | | |
| | Sample ID No | | |
| | Type of sample | | |
| | Date of Collection | | |
| | Time of sample collection | | |
| | Place of sample collection | | |
| | Description of sample | | |
| | Analysis required | | |
| | Name & signature of the sample collector | | |
| | Date of lab test performed | | |
| | Name of pathogen identified | | |
| | **Diagnostic method by which pathogen identified:**<br>● Culture and biochemical tests<br>● PCR<br>● RT-PCR<br>● Toxin assay<br>● Kit-based detection | | |
| | **Antibiotic Sensitivity Report** | | |
| | Sensitive to | Resistant to | |

**Supplementary Table 3:** Overview of data elements of market survey

| | |
|---|---|
| **Food item collection from markets, street foods, food stalls, restaurants and other food handling establishments** | Sample ID No |
| | Type of sample |
| | Date of Collection |
| | Place of sample collection |
| | Name of contact person/shop/participant |
| | Address of contact person/shop/participant |
| | Phone number of contact person/shop/participant |
| **Information on personal hygiene of food handler** | Are there handwashing facilities in the market? If yes, describe |
| | How many times do you wash hands? |
| | Cutting of nail |
| | Good personal Hygiene (observed by observer) |
| | Is the market cleaned regularly? |
| | Is the market cleaned regularly? |
| | Is the market properly zoned to avoid cross- |

| | | |
|---|---|---|
| | contamination? For example, live animals and raw foods of animal origin should be separated from<br>ready-to-eat foods. | |
| | Are there any cold storage facilities? | |
| | Is food exposed to flies? | |
| | Overall surrounding Clean & healthy | |
| **Information on the surrounding environment (street foods, food stalls, restaurants and other food handling establishments)** | Do vendors wash their hands before preparing and serving food? | |
| | Is clean water used for washing dishes? | |
| | How will you describe the immediate environment where food is prepared? | |
| | Is food well-covered? | |
| | Is food exposed to flies? | |
| | Is the food at ground level? | |
| | Original Source/supplier of animal /vegetable other food product | |
| | Are there any cold storage facilities? | |
| | Time for preservation | |
| | Overall surrounding Clean & healthy | |
| **Lab Report** | **Baseline Data (0 day)** | |
| | Sample ID No | |
| | Type of sample | |
| | Date of Collection | |
| | Time of sample collection | |
| | Place of sample collection | |
| | Description of sample | |
| | Analysis required | |
| | Name & signature of the sample collector | |
| | Date of lab test performed | |
| | Name of pathogen identified | |
| | **Diagnostic method by which pathogen identified:**<br>● Culture and biochemical tests<br>● PCR<br>● RT-PCR<br>● Toxin assay<br>● Kit-based detection | |
| | **Antibiotic Sensitivity Report** | |
| | Sensitive to | Resistant to |

**Supplementary Table 4:** Overview of Data Elements of poultry/farm/slaughter house surveillance

| | |
|---|---|
| **Case Enrollment Details** | Facility visited (Poultry/animal farm/ slaughterhouse etc.) |
| | Sample ID No (Naming convention): STATE/DIST/CAT/SUB/Seq |
| | Type of sample |
| | Date of Collection: DD/MM/YYYY |
| | Source from where the animals brought to the facility |
| | Date of animal brought to the facility |
| | Total number of animals in that facility |
| | Name of contact person/animal handler/owner of animal farm/poultry etc. |
| | Address of contact person/shop/slaughterhouse/poultry farms |
| | Phone number of contact person/shop/participant |
| **Information on personal hygiene of animal handler** | Are there hand washing facilities in the slaughterhouse/ poultry farms |
| | How many times do you wash your hands? |
| | Cutting of nails |
| | Good personal Hygiene (observed by the observer) |
| | Are you using disposable gloves during animal handling? |
| | Is the animal handler suffering from any skin disease/ fever/ diarrhea or any other disease? |
| **Information of farm/animal husbandry/ slaughterhouse etc.** | Is the slaughterhouse/poultry farm cleaned regularly? |
| | How many animals placed in a room/cage? |
| | Is there any clean water facility? |
| | Is there any regular animal vaccination facility or animal is vaccinated? |
| | Turnaround time or average time of animal keeping in this facility? |
| | Condition of animal |
| | Total number of animals sampled |
| | Has the animal ever been contacted communicable disease? |
| | Types of Animal Feed |
| | Method of Feeding the animals in farm/slaughter house |
| | Is animals given any antibiotics as prophylaxis |
| | Milch animals: Is animal body parts clean before |

| | |
|---|---|
| | obtain milk? Udder is cleaned before milking |
| | Time of preservation of milk, egg, etc |
| | Poultry: Is eggs cleaned from animal faeces before storage/transport? |
| | Is there any regular health check-up facility by veterinarian? |
| | Is there any waste disposal facility |
| | Is animal given any wash before slaughtered ? |
| | Cleaning of slaughtering equipment done using |
| | Facility for regular disinfection of slaughtering equipment |
| | Time for preservation of raw meat |
| | Are there any cold storage facilities? |
| | (If Yes) Transportation time to the nearest facility |
| | Processing area cleanliness |
| | Chiller/Freezer room cleanliness |
| | Does the slaughterhouse/ has a disposal treatment facility? |
| | Is the facility properly zoned to avoid cross-contamination? (For example, live animals and raw foods of animal origin) |
| | Is raw meat exposed to flies/animal urine/faeces/other contaminant? |
| | Is overall surrounding clean & healthy? |
| | Does butcher use gloves while handling raw meat? |
| | Is butcher suffering from any diseases? |
| **Details of sample collection and laboratory investigations** | a) Routine testing (at site lab)<br>b) Referred testing (Laboratory referred testing, NICED/RMRC) |
| | Date of sample collection for which the results of the pathogens were positive. |
| | Specimen type collected for diagnosis of pathogens |
| | Date of identification of the pathogen |
| | Type of Investigation done |
| | Antibacterial Agent tested |
| **Pathogen distribution** | Bacterial pathogen type |
| | Viral pathogen type |

**Supplementary Table 5: Overview of Data Elements of Outbreak Investigation**

| | |
|---|---|
| Date | |
| Name of the person completing form | |
| **Information on the person reporting disease outbreak** | Place of outbreak |
| | Name of contact person |

| | |
|---|---|
| | Address of contact person |
| | Phone number of contact person |
| **Information on household hygiene & food handling practices** | |
| **Information on water supply (accessibility, adequacy, quality, storage)** | How distant is water collection points from where people live? |
| | How much water is available per person a day (for cooking and personal hygiene)? |
| | Where do people get water for washing their hands and cooking food? |
| | How is water transported? |
| **Sanitation** | Is there any possibility of contamination during storage and transportation due to the containers used? |
| | Do people have access to sanitation facilities? Which facilities? |
| | If so, are they sufficiently used? |
| | Where is the waste disposed of? |
| | Are there handwashing facilities in households? |
| | How many times do you wash hands? |
| | How will you describe the immediate environment where food is prepared? |
| | Is food cooked at home? |
| | If so, where is food cooked? |
| | What are the types of food items commonly consumed? |
| | Are there any traditional dishes containing raw foods? |
| | Sources of domestic food supply? |
| | What precautions are taken to avoid poor handling of food? |
| | Is food well-covered? |
| | Is food exposed to flies? |
| | Are there any cold storage facilities? |
| | Animal rearing |
| | Type of animals |
| | Overall surrounding Clean & healthy |

| | | |
|---|---|---|
| **Information on Disease Outbreak** | | |
| **CRF NO** | Date: DD/MM/Year | Name of the person completing the form |
| **Hospital Name** | | |
| **Information of the patient** | Name | |
| | Age | |

<table>
<tr><td rowspan="3"></td><td colspan="2">Gender</td></tr>
<tr><td colspan="2">Ethnicity</td></tr>
<tr><td colspan="2">Address & Phone number</td></tr>
<tr><td rowspan="21">Clinical details</td><td colspan="2">Date and time of onset of symptoms</td></tr>
<tr><td colspan="2">Date and time when symptoms stopped</td></tr>
<tr><td colspan="2">Predominant symptoms</td></tr>
<tr><td>Symptom</td><td>Frequency/Duration</td></tr>
<tr><td>Diarrhea</td><td></td></tr>
<tr><td>Blood in stool</td><td></td></tr>
<tr><td>Vomiting</td><td></td></tr>
<tr><td>Nausea</td><td></td></tr>
<tr><td>Fever/feverish</td><td></td></tr>
<tr><td>Abdominal Pain</td><td></td></tr>
<tr><td>Others</td><td></td></tr>
<tr><td colspan="2">Doctor consulted? (if yes, provide name and details such as the type of treatment, duration of illness, and names of antibiotics if any)</td></tr>
<tr><td colspan="2">Date of admission</td></tr>
<tr><td colspan="2">Date of discharge</td></tr>
<tr><td colspan="2">Diagnosis available?</td></tr>
<tr><td colspan="2">Treatment details</td></tr>
<tr><td colspan="2">Antibiotic details (if taken)</td></tr>
<tr><td colspan="2">Duration of treatment</td></tr>
<tr><td colspan="2">Outcome</td></tr>
<tr><td colspan="2">Laboratory specimen taken? (if yes, provide details)</td></tr>
<tr><td>Nature of the specimen</td><td>Lab results</td></tr>
<tr><td rowspan="9">Exposure history of the patient</td><td colspan="2">Suspect food?</td></tr>
<tr><td colspan="2">Date and time of exposure to an implicated food or event (if known)</td></tr>
<tr><td colspan="2">Source of food</td></tr>
<tr><td colspan="2">Suspect meal, event, place? (if yes, describe; provide, name, date, address, phone) contact with people with similar clinical signs and symptoms</td></tr>
<tr><td colspan="2">Persons attending suspect meal/event</td></tr>
<tr><td>Ill/well</td><td>Address & contact no.</td></tr>
<tr><td colspan="2">Recent farm visits</td></tr>
<tr><td colspan="2">Contact with animals</td></tr>
<tr><td colspan="2">Working as a food handler</td></tr>
</table>

| | | | |
|---|---|---|---|
| | Attending or working in a school, Child-care facility, medical facility | | |
| | Chronic illness, immunosuppression, pregnancy | | |
| | Recent changes in medical history, regular medications | | |
| | Allergies, recent immunizations | | |
| | Information on recent travel (domestic and international) | | |
| **Food History** | **Have you eaten any of the following**? | **Amount** | **Source** |
| | Poultry | | |
| | Pork | | |
| | Beef | | |
| | Lamb | | |
| | Chicken | | |
| | Dried smoked meat | | |
| | Crab chutni | | |
| | Dried smoked fish | | |
| | Fish chutni | | |
| | Gungdruk | | |
| | Cauliflower | | |
| | Carrots | | |
| | Green salad | | |
| | Other salads | | |
| | Roast potatoes | | |
| | Fried potatoes | | |
| | Mayonnaise | | |
| | Milk | | |
| | Stored food | | |
| | Ambient | | |
| | Refigerated | | |
| | Other | | |
| **Lab Report** | **Baseline Data (0 day)** | | |
| | Sample ID No | | |
| | Type of sample | | |
| | Date of Collection | | |
| | Time of sample collection | | |
| | Place of sample collection | | |
| | Description of sample | | |
| | Analysis required | | |
| | Name & signature of the sample collector | | |
| | Date of lab test performed | | |

| | | |
|---|---|---|
| | Name of pathogen identified | |
| | **Diagnostic method by which pathogen identified:**<br>● Culture and biochemical tests<br>● PCR<br>● RT-PCR<br>● Toxin assay<br>● Kit-based detection | |
| | **Antibiotic Sensitivity Report** | |
| | Sensitive to | Resistant to |
